# Knowledge, awareness and recommendation on micronutrition during pregnancy—a survey of healthcare providers

**DOI:** 10.1007/s00404-025-08111-6

**Published:** 2025-07-10

**Authors:** Anna Elisabeth Hentrich, Doerthe Brueggmann, Eileen Deuster, Anne Kristina Kämpf, Lukas Jennewein, Wiebke Schaarschmidt, Frank Louwen, Samira Catharina Hoock

**Affiliations:** Department of Obstetrics and Perinatal Medicine, University Hospital, Goethe University Frankfurt, 60590 Frankfurt, Germany

**Keywords:** Micronutrient pregnancy, Supplementation, FIGO nutrition checklist

## Abstract

**Background:**

The market for dietary supplements targeting women of reproductive age and pregnant individuals is rapidly expanding. Despite accumulating evidence regarding the benefits of specific micronutrients during pregnancy, there is considerable variability in international and national guidelines, compounded by knowledge gaps among healthcare professionals.

**Objective:**

This study aimed to assess the knowledge, attitudes, and counseling practices of midwives and physicians in Germany concerning micronutrient supplementation during pregnancy.

**Methods:**

A cross-sectional online survey was conducted among healthcare professionals in Frankfurt and surrounding regions in April to May 2024. A newly developed 24-item questionnaire was utilized to gather demographic data, assess counseling practices related to micronutrients and knowledge concerning of the roles of folic acid, vitamin D, vitamin B12, and omega-3 fatty acids in fetal development. Data were analyzed using descriptive statistics and Fisher's exact test (*p* < 0.05).

**Results:**

Of the 360 individuals who accessed the survey, 107 completed it (33 midwives, 72 physicians). While 96.8% recommended supplementation during pregnancy, only 48.1% rated their knowledge as moderate. Folic acid was most frequently recommended supplement (78.7%), followed by omega-3 fatty acids (68%) and vitamin B12 (notably for vegan diets, 96.1%). Gaps in knowledge were identified, particularly regarding the biochemical forms of folate (34.5% unaware), sources and function of omega-3 fatty acids (20% lacked knowledge), and the role of vitamin B12 in fetal development (19% unaware). Only 41.8% explicitly recommended vitamin D, despite strong evidence of its importance. The majority of respondents expressed a strong interest in further education (91.0%).

**Conclusion:**

Although healthcare professionals are generally engaged in counseling on prenatal supplementation, substantial knowledge gaps and inconsistent practices persist, particularly regarding newer recommendations beyond folic acid. To improve maternal and fetal health outcomes, there is a pressing need for improved educational initiatives and the broader implementation of tools such as the FIGO nutrition checklist.

## Introduction

The market for dietary supplements has expanded substantially in recent years, with an increasing array of products targeting pregnant women and those of reproductive age. Advertisements for supplements tailored to specific life stages—such as for individuals in the fertile phase, women over the age of 35, those with polycystic ovary syndrome (PCOS), and various stages of pregnancy—are widespead. The diversity in the ingredients and prices of these products is notable, as dietary supplements do not undergo the same rigorous scrutiny as pharmaceuticals. Data on the appropriate intake of micronutrients prior to conception and during pregnancy are expanding, with notable contribution from Cochrane reviews on individual micronutrients [1–3] and guidelines established by the World Health Organization (WHO) [4, 5]. The International Federation of Gynecology and Obstetrics (FIGO) has developed a checklist to assess the nutritional status of pregnant women [6, 7]. While international guidelines generally endorse the recommendation of folic acid supplementation, there is considerable inconsistency regarding other micronutrients. A Delphi expert consensus at the FIGO Congress in 2023 emphasized the necessity of supplementation with vitamin D, omega-3 fatty acids, iodine, and iron in addition to folic acid. Notably, the U.S. Dietary Guidelines for 2020–2025 have included recommendations for pregnant women and children for the first time.

In Germany, the German Nutrition Society (DGE) recommends supplementation with 400–800 µg of folic acid, 100–150 µg of iodine, and 200 mg of DHA (docosahexaenoic acid) for individuals who do not consume sufficient amounts of fatty fish. Furthermore, it is advised to optimize vitamin D levels [8]. Since spring 2024, newborn screening in Germany has included testing for vitamin B12 deficiency due to the inreasing incidence of such deficiencies among vegan and vegetarian mothers [9].

Given the wide array of available supplements and the growing body of evidence supporting the importance of adequate intake and potential supplementation of vitamin D, omega-3 fatty acids, and vitamin B12—alongside folic acid—during pregnancy, midwives and obstetricians serve a pivotal role in educating patients and identifying potential nutrient deficiencies. As advocated by FIGO, nutritional counseling and weight management should be integrated into every prenatal consultation [10].

The objective of this study was to assess the knowledge and practices of healthcare professionals regarding micronutrient supplementation during pregnancy and to evaluate the specific recommendations provided to pregnant women.

## Materials and methods

### Study population and data collection

This cross-sectional observational study was conducted in Frankfurt, Germany and its surrounding areas to assess the knowledge and recommendations of midwives and obstetricians regarding supplement use during pregnancy. Data were collected via an online questionnaire (accessible via a link or QR code), distributed through social media groups in April and May 2024. The survey was conducted on an anonymous platform (SoSci Survey) with approval of the ethics committee (ethic number 2024–1732).

### Questionnaire

No validated German questionnaire specific to this topic was identified in the literature; therefore, a new quantitative questionnaire was developed (available upon request from the corresponding author). The questionnaire was pre-tested by five physicians and psychologists. The participants completed the survey in approximately 7–9 min.

The questionnaire consisted of 24 items divided into three sections:

- Demographic data and sources of information (9 items)
- Specific micronutrient recommendations provided to patients (10 items)
- Assessment of healthcare providers' knowledge regarding the impact of folic acid, vitamin B12, vitamin D, and omega-3 fatty acids on fetal development (5 items)

All variables were categorized into dichotomous outcome variables or nominal independent variables. The survey used multiple-choice questions allowing respondents to select one or more answers.

Independent variables included occupation, years of professional experience, age (nominal), and general recommendations for the supplementation of folic acid, omega-3 fatty acids, vitamin D, and vitamin B12. Knowledge of the micronutrients was evaluated through questions regarding whether the nutrients could be adequately obtained from a normal diet and their perceived benefits for fetal development. This was assessed with using a „which statement is correct?" format.

Additionally, participants were asked if they inquired about their patients' dietary habits (yes/no) and where they obtained their information regarding pregnancy nutrition (yes/no).

### Data analysis

Descriptive statistics were used for data analysis with percentage, numbers, standard deviation and means, employing SPSS for Apple Version 29.0.2.0 (20). Associations between professional groups were evaluated using Fisher’s exact Test with a significance level set at 5% (*p* < 0.05).

## Results

Out of the 360 individuals who accessed the surey, 107 complete it.

## Demographic results

A total of 33 midwives (7.5% self-employed) and 72 physicians participated. Among the physicians, 21.5% were assistant doctors, 15.9% specialists in outpatient settings, and 29.9% specialists working in hospitals. Two participants did not respond to this question.

**Fig. 1:**
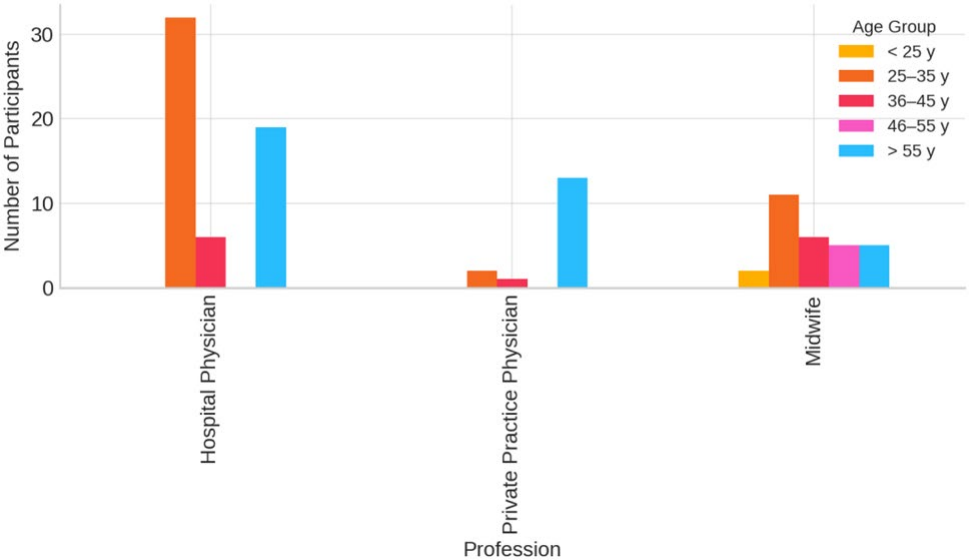
Age Distribution by Profession Distribution of participants' age groups stratified by profession

The age distribution was as follows: 41.9% were between 25 and 35 years old, and 18.6% were over 46 years old. On average, participants had 12.2 years of professional experience (SD ± 9.028) (Fig. 1).

A majority (92.4%) reported receiving information about nutrition during pregnancy. The primary sources of information included professional literature, the internet, and formal education (studies or continuing education), with one-third of respondents obtaining information from colleagues.

Nearly all participants (96.8%) recommended dietary supplements during pregnancy, with folic acid being the most frequently recommended (78.7%). The majority of respondents (60.6%) recommended a combination supplement. However, 7.4% of participants were unaware of the reasons for recommending supplementation, and 5.1% indicated a lack of interest in the topic, and 3.8% cited time constraints as a reason insufficient counseling.

## Specific micronutrient recommendations

Three-quarters of the healthcare professionals inquired about their patients' dietary habits (74.0%). A significant majority (96.1%) recommended vitamin B12 supplementation for vegan pregnant women.

Folic acid was recommended by 53.2% of respondents for 3 months prior to conception. A majority (41.4%) recommended folic acid supplementation in general, without distinguishing between different biochemical forms. Furthermore, 34.5% of participants were unaware of the differences between folic acid and its methylated form, folate.

Omega-3 supplementation was recommended by 68% of respondents, with a similar proportion recommending either a combination supplement containing DHA (200 mg) or a specific omega-3 supplement that included both DHA and EPA. 17.3% recommended obtaining omega-3 through dietary intake alone. 20% of respondents reported lacking knowledge regarding omega-3 supplementation. There was no significant difference between the professional groups regarding the recommendation for omega-3 intake (*p* = 1.00).

**Fig. 2:**
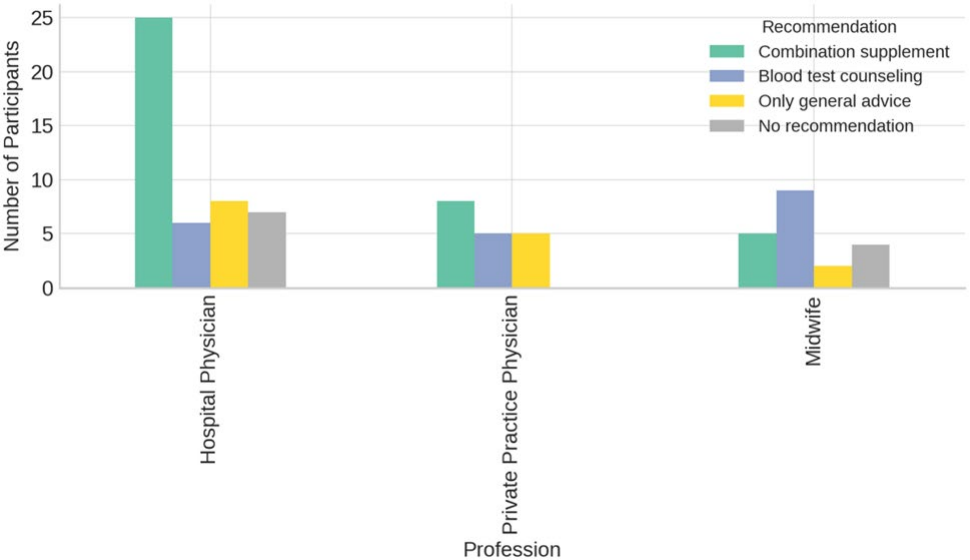
Recommendations for Vitamin D Supplementation by Profession Reported recommendations for Vitamin D supplementation among different healthcare professions

Vitamin D was not recommended by 13.9% of respondents, while 25.3% recommended supplementation only after assessing the vitamin D serum level. 11.4% endorsed the general use of a vitamin D supplement. The majority (49.3%) recommended a combination supplement for vitamin D supplementation during pregnancy. There were significant differences between midwives and physicians regarding the various statements on vitamin D recommendation (*p* = 0.028) (Fig. 2).

## Assessment of healthcare providers’ knowledge

The respondents generally recognized the insufficient intake of folic acid (75.9%) and vitamin D (83.5%) from food alone. According to 48.1% of participants, omega-3 could be adequately obtained from a mixed diet, while 72.2% believed this was the case for vitamin B12.

The majority (89.9%) acknowledged the importance of folic acid for fetal neural development, while 80.8% associated vitamin D with fetal bone development. However, 17.7% of participants were unaware of the role of vitamin D and omega-3 in fetal development, and 19% were unaware of the function of vitamin B12. A significant proportion of participants (59.5%) recognized the role of omega-3 in fetal brain development, while 78.5% acknowledged its importance for neural development (Table 1).

**Table 1:** Summary Table of Key Findings: micronutrient, percentage of healthcare providers aware of its function, what the supplement aims to improve, evidence of effect (RR with 95% CI, RR values are cited from recent meta-analyses and major reviews where available)

Micronutrient	% health care providers Aware of Function	Clinical Benefit	Evidence of Effect (RR, 95% CI)
Folic Acid	89.9%	Prevents neural tube defects	0.28 (0.15–0.52)
Vitamin D	80.8%	Supports bone development, reduces preeclampsia risk	0.48 (0.30–0.79)
Vitamin B12	60.8%	Prevents neural tube defects supports neurodevelopment	Less data
Omega-3 Fatty Acids	78.5%	Supports neural/brain development, reduces preterm birth	0.89 (0.81–0.97) (preterm birth < 37 weeks) 0.58 (0.44 − 0.77) (preterm birth < 34 weeks)

**Fig. 3:**
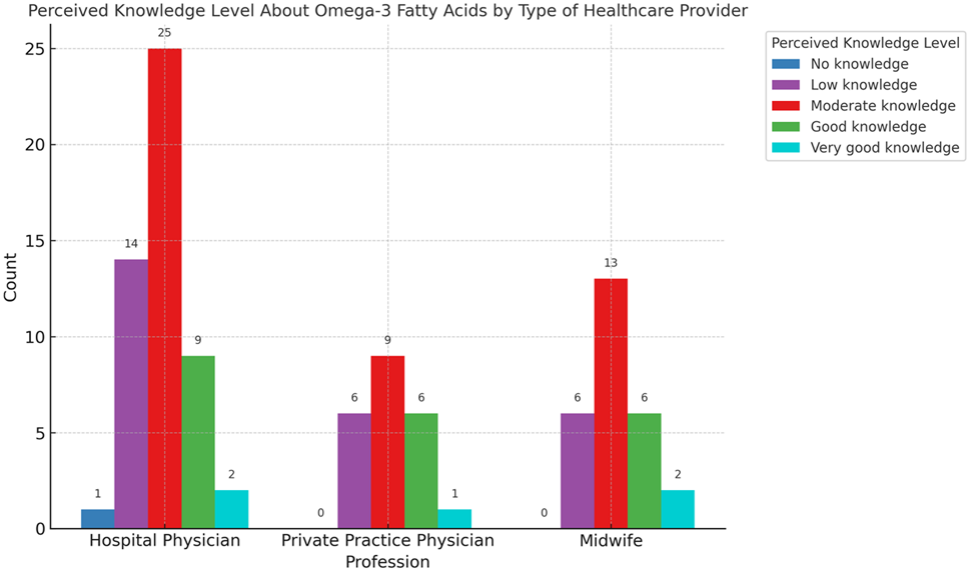
Distribution of perceived knowledge levels about omega-3 fatty acids among pregnant women, categorized by type of healthcare provider consulted

Regarding self-assessment, 48.1% of respondents rated their knowledge of prenatal supplementation as moderate, while 25.3% considered themselves to have limited or no knowledge (Fig. 3). The self-assessment of knowledge levels among the professional groups was not significantly different (*p* = 0.251). A vast majority (91.0%) expressed interest in further educational resources.

## Discussion

The survey conducted among physicians and midwives indicates that participants are engaged with maternal nutrition and supplementation during pregnancy, and they recommend dietary supplements to their patients. Folic acid, in accordance with WHO and DGE guidelines [4, 8], was the most frequently recommended supplement. This finding aligns with the study by Buhling et al. [11]. In accordance with international recommendations, supplementation is most commonly recommended preconceptionally, particularly when the respondents are already familiar with their patients [4] [12, 13]. Furthermore, 80% of the respondents acknowledge that adequate folic acid intake through diet alone is insufficient, as shown in the National Consumption Study II [12].

The impact of folic acid on fetal neural development and the prevention of neural tube defects is well-established and recognized by 89% of the participants [3]. Supplementation reduces the risk of neural tube defects. De-Regil et al. reported a risk reduction (RR) of 0.31 (95% CI 0.17–0.58) [1]. Methylenetetrahydrofolate reductase (MTHFR) catalyzes the conversion of folic acid to methylated folate [14]. Polymorphism of MTHFR are associated with varying enzyme activity leading to lower serum folate levels [14, 15]. International recommendations currently recommend the intake of 400 µg of folic acid daily to prevent neural tube defects [3, 4]. Various companies promote products containing methylated folate as a more bioavailable form, prompting women to consult their gynecologists for further questions. However, research demonstrating the benefits of folate intake remains limited and 38% of respondents are even uninformed about the different biochemical forms of folic acid. This highlights the importance of enhanced education for health care professionals.

In addition to folic acid, vitamin B12 plays a pivotal role in preventing neural tube defects [16, 17]. It is also involved in bone and energy metabolism, as well as in cell division [18]. A deficiency of vitamin B12 during pregnancy can lead not only to the aforementioned malformations but also to intrauterine growth restriction, reduced muscle mass, and an increased risk of chronic diseases in later life [19]. Maternal deficiency is associated with an increased risk of gestational diabetes [19, 20], and lower vitamin B 12 levels were observed in women with preeclampsia [21]. Vitamin B12 is primarily found in animal products, with deficiency is more common in individuals following vegan and vegetarian diets [19]. Additionally, vitamin B12 absorption requires the gastric intrinsic factor, making women who have undergone bariatric surgery prone to deficiency [19]. Our survey revealed that 96% of participants were aware of the need for vitamin B12 supplementation in vegan diets. However, only 74% of professionals inquire about their patients' dietary habits. Studies indicate that 5% of Canadian women and 20% of pregnant women in the US are vitamin B12 deficient [22, 23]. The incidence of vitamin B12 deficiency in neonates born to women in Germany stands at 1 in 5355, prompting the expansion of the newborn screening (NBS) in Germany to include vitamin B12 starting in 2024 [9]. In our survey, 19% of respondents were unaware of the role of vitamin B12 in fetal development.

A good level of knowledge regarding folate supplementation was also demonstrated by Allish et al. in their survey of Australian midwives [24]. Similar to our questionnaire, however, a decline in knowledge regarding vitamin B12 was observed, as well as a gap in discussions with patients about their nutrition. As in our questionnaire, a difference was also found between those working in hospitals and in outpatient settings, which can be explained by the different daily routines in these environments.

Vitamin D, classified as a hormone, undergoes metabolism in a sunlight-dependent manner. The WHO and clinical practical guidelines in the USA recommend vitamin D supplementation during pregnancy, depending on sunlight exposure. The German Nutrition Society recommends a vague 20 µg/day of vitamin D in cases of insufficient or absent sunlight exposure [8]. Whether this dosage is adequate, particularly during the winter months, is inconsistently discussed and recommended in the literature [1]. There is no standardized recommendation for serum vitamin D testing prior to supplementation or a daily dosage recommendation [1, 5, 25]. Vitamin D deficiency during pregnancy varies by country, with prevalence rates ranging from 20 to 85% [26].

Vitamin D, along with calcium, is essential for bone health and is transplacentally transferred to the fetus [26, 27]. Deficiency can lead to low birth weight and disorders in fetal bone development. Maternal vitamin D deficiency is associated with an increased risk of insulin resistance and gestational diabetes, while supplementation has shown to reduce the risk of preeclampsia [1, 26, 28, 29]. Supplementation during pregnancy is associated with a reduced risk of preeclampsia (RR 0.48, 95% CI 0.30–0.79) and low birth weight (RR 0.55 95% CI 0.35,0.87) [2].

Our survey revealed that although the majority of experts recognize the benefits of vitamin D supplementation, only 41.8% explicitly recommend it, a finding consistent with literature reports [11]. While 49.3% recommend vitamin D supplementation only in combination with other supplements, plasma vitamin D testing is performed in 25.3% of cases. Given the limited sunlight exposure in Germany during winter and evidence supporting the reduction of gestational diabetes and preeclampsia risks, the recommendation rate for vitamin D supplementation should be improved.

Research indicates that omega-3 supplementation during pregnancy reduces the risk of preterm birth (RR 0.89, 95% CI 0.81–0.97 for < 37 weeks and RR 0.58, 95% CI 0.44 − 0.77 for < 34 weeks) [4–6], which is attributed to its observed effects on placental tissue [32]. Additionally, omega-3 contributed to fetal neural development [2]. The WHO and DGE recommend 200 mg of DHA daily during pregnancy, which can also be obtained from consuming fatty fish [8]. Approximately 50% of the participants recognized the benefits of omega-3 for fetal development. Soliman et al. also published an expert consensus by Egyptian gynecologists on micronutrient intake, in which 86% recommended omega-3 supplementation during the third trimester [33]. However, gaps in knowledge persist among healthcare providers, particularly regarding the distinction between DHA and EPA, as well as the potential benefits of higher doses of omega-3 supplementation. This was also noted in the study by Buhling et al. [11].

In conclusion, our survey suggests that healthcare professionals do not consider themselves well-informed on prenatal supplementation and express a desire for further education in this field [11]. It should be noted that most hospital-based physicians in Germany are not routinely involved in prenatal care, and our sample included a large proportion of early-career professionals (21.5%). These factors may limit the generalizability of our findings and could partially explain the observed knowledge gaps However, providing counseling in clinical practice necessitated significant time investments, as acknowledged by our participants and noted as a shortcoming in the Nutrition Checklist developed by FIGO [6].

## Conclusion

Given the increasing prevalence of vegan and vegetarian diets, along with the consumption of nutrient-poor processed foods, and rising rates of obesity and gestational diabetes, the role of healthcare professionals in guiding pregnant women on micronutrient supplementation is crucial. Tools such as the FIGO checklist for efficient patient consultation and risk assessment should be more widely disseminated, with a particular focus on providing additional educational resources for healthcare workers.

## Limitations

This study is subject to several limitations. First, selection and response bias may have influenced the results, as individuals with a particular interest in nutrition may have been more likely to participate. Second, the use of self-reported data introduces the potential for recall inaccuracies and social desirability bias. Third, the geographic and professional scope of the sample—primarily from Frankfurt, and with a high proportion of early-career professionals—may limit the generalizability of the findings to the wider population of healthcare providers. These limitations should be considered when interpreting the results.

## Data Availability

No datasets were generated or analysed during the current study.
